# Overexpression of *GhABF3* increases cotton(*Gossypium hirsutum* L.) tolerance to salt and drought

**DOI:** 10.1186/s12870-022-03705-7

**Published:** 2022-06-29

**Authors:** Haijun Zhang, Lili Mao, Ming Xin, Huixian Xing, Yanan Zhang, Jing Wu, Dongli Xu, Yiming Wang, Yongqi Shang, Liming Wei, Mingshuo Cui, Tao Zhuang, Xuezhen Sun, Xianliang Song

**Affiliations:** 1 State Key Laboratory of Crop Biology/Agronomy College, Shandong Agricultural University, Taian, China; 2 College of Tropical Crops, Hainan University, Haikou, China; 3 Shenxian Agriculture and Rural Bureau, Liaocheng, China

**Keywords:** *GhABF3*, Drought stress, Salt stress, VIGS, Overexpression

## Abstract

**Background:**

Plants suffer from various abiotic stresses during their lifetime, of which drought and salt stresses are two main factors limiting crop yield and quality. Previous studies have shown that abscisic acid (ABA) responsive element binding protein (AREB)/ ABRE binding factors (ABFs) in bZIP transcription factors are involved in plant stress response in an ABA-dependent manner. However, little is known about the properties and functions of AREB/ABFs, especially *ABF3*, in cotton.

**Results:**

Here, we reported the cloning and characterization of *GhABF3*. Expression of *GhABF3* was induced by drought,salt and ABA treatments. Silencing of *GhABF3* sensitized cotton to drought and salt stress, which was manifested in decreased cellular antioxidant capacity and chlorophyll content. Overexpression of *GhABF3* significantly improved the drought and salinity tolerance of Arabidopsis and cotton. Exogenous expression of *GhABF3* resulted in longer root length and less leaf wilting under stress conditions in *Arabidopsis thaliana*. Overexpressing *GhABF3* significantly improved salt tolerance of upland cotton by reducing the degree of cellular oxidation, and enhanced drought tolerance by decreasing leaf water loss rate. The increased expression of *GhABF3* up-regulated the transcriptional abundance of downstream ABA-inducible genes under salt stress in Arabidopsis.

**Conclusion:**

In conclusion, our results demonstrated that *GhABF3* plays an important role in plant drought and salt tolerance. Manipulation of *GhABF3* by biotechnology might be an important strategy to alter the stress resistance of cotton.

**Supplementary Information:**

The online version contains supplementary material available at 10.1186/s12870-022-03705-7.

## Background

With the rapid development in industrialization and urbanization, more and more environmental problems are threatening the growth of crops, such as global warming, soil salinization, desertification and so on [1]. As a source of natural fiber and edible oil, cotton is an important economic crop in the world. Although it has certain adaptability to salinity and drought, its yield and fiber quality are still restricted by the harsh living environment [2, 3]. Therefore, it is still of great significance to further improve the salinity and drought tolerance of cotton.

In response to environmental stresses, plants have evolved complex signaling pathways, namely receptors, secondary messengers, phytohormones and signal transducers [4, 5]. ABA is a very important plant hormone, which has an obvious inhibitory effect on seed germination and seedling growth [6]. Previous studies have shown that ABA can promote stomatal closure, leaf and fruit shedding, and also has a very important relationship with seed dormancy [7–9]. The ABA content in plants rises rapidly and forms a pathway to inhibit its degradation under stress [10], showing that ABA plays an important role in plant response to stress.

Under stress conditions, the content of ABA increases, and protein phosphatase 2C (PP2C) is recognized by its receptor protein PYR/PYL/RCARs to form a receptor complex, thereby blocking PP2C mediated dephosphorylation of sucrose non-fermenting 1-related protein kinase 2 (SnRK2) and thereby activating SnRKs [11–13]. The SnRKs phosphorylate downstream members, including transcription factors (TFs) such as AREB, and finally activate the expression of target genes in response to ABA signal, such as late embryogenesis abundant (LEA) protein gene and related regulatory genes [14].

AREB/ABF transcription factors belong to the A subfamily of bZIP transcription factors, and their functions involve plant response to stress and ABA signals [15–18]. AREB/ABF transcription factors contain four conserved domains, three (C1, C2, and C3) at the N–terminal half and one (C4) at the C–terminus. Within C1, C2, C3 and C4 domains are well-conserved consensus phosphorylation sites for protein kinases. These domains contain RXXS/T conserved sequence and can be phosphorylated by serine/threonine protein kinase [19]. The AREB/ABF transcription factors not only regulate plant growth and development, but also play an important role in plant response to abiotic stresses such as drought, low temperature and high salt concentration. Previous studies showed that *AtABFs* could promote ABA-mediated chlorophyll [20–22]degradation and leaf senescence [23]. Overexpressing *NtABF* induced the up-regulation of reactive oxygen species (ROS) scavenging gene and improved the antioxidant ability of tobacco [24]. Overexpression of *AtABF3* enhanced tolerance to various abiotic stresses and reduced leaf size in alfalfa [25]. Heterologous expression of *DcABF3* altered stomatal density and reduced ABA sensitivity and improved drought resistance in Arabidopsis [26]. The silencing of *RhABF2* reduced rose (*Rosa hybrid*) dehydration tolerance and disrupted Fe homeostasis in rose petals during dehydration [27]. High salt concentration inhibited the expression of *GhABF2* in cotton, but the salt tolerance of cotton overexpressing *GhABF2* was improved [21]. Overexpression of *GhABF2D* and *AtABF3* in *G. hirsutum*, respectively, improved the drought resistance of cotton [28].

Based on the results of previous studies, ABF transcription factors have functional diversity and might have distinct mechanisms of action in different crops. A total of eight *ABF* genes were previously identified in cotton, among which *ABF2A* and *ABF2D* have been characterized in detail [21, 28, 29]. This study identified a novel AREB/ABF transcription factor, *GhABF3* (XM_016888701), in *G. hirsutum*, demonstrating that it played a critical role in ABA-mediated drought and salt tolerance in cotton. These results deepened our understanding of the function and mechanism of *GhABF3* gene in response to drought and high salt stress.

## Results

### Bioinformatics analysis of *GhABF3*

**Fig. 1:**
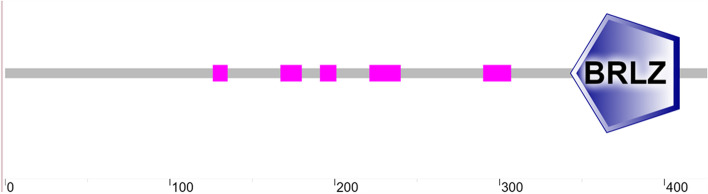
GhABF3 domain identification. Pink blocks represented low complexity regions, gray bars represented full-length amino acid sequences, and blue blocks were BRLZ (basic region leucin zipper)

We cloned and isolated *GhABF3* gene from upland cotton Han682. The full length of the gene was 2898bp, with three introns separating four exons. The full length of CDS spanned 1281bp coding 426 amino acids. The ABF3 protein contained a typical bZIP domain BRLZ at the C terminal (Fig. 1), and was considered to be a typical bZIP protein. The molecular weight of the protein was 46321.92D, and the theoretical isoelectric point was 9.56. The instability index of the ABF3 protein was 56.41, and the hydrophilic mean value was -0.713, indicating that it was an unstable hydrophilic protein. By cell-ploC prediction, subcellular localization was located in the nucleus. The protein contained 31 Serine, 18 Threonine and 1 Tyrosine phosphorylation site, without signal peptide and cleavage site. The secondary structure of ABF3 protein contained 31.46% alpha helix, 10.56% extended strand, 3.29% beta turn and 54.69% random coil.

### Expression pattern of *GhABF3*

**Fig. 2:**
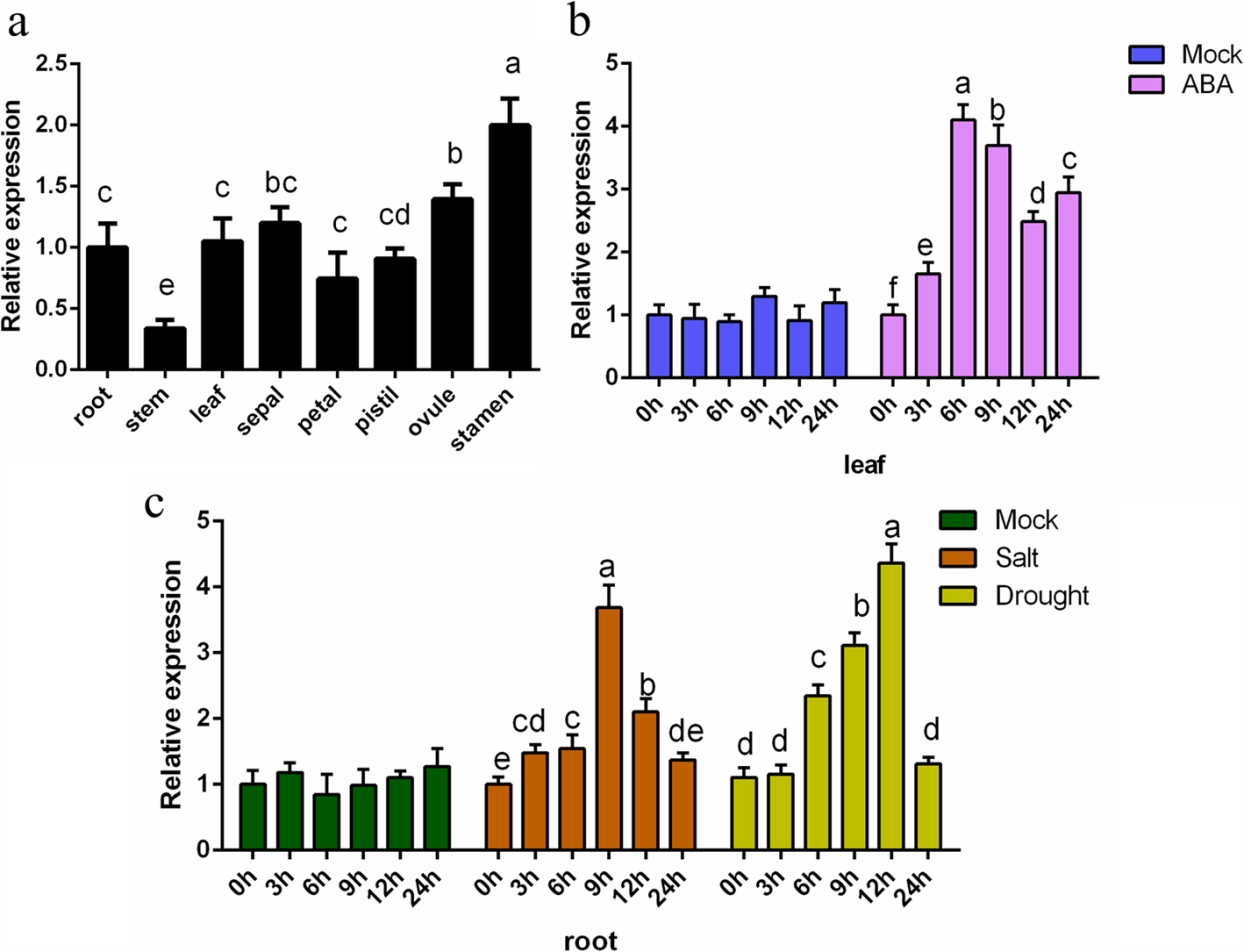
Expression patterns of *GhABF3.* **a** *GhABF3* transcript abundance in different organs. **b** Expression pattern of *GhABF3* in cotton leaves under 500μM ABA stressed. The sampling time for each sample was 0, 3, 6, 9, 12 and 24h after treatment. **c** Gene expression under 250mM NaCl and 10% PEG6000 stresses. The sampling time for each sample was 0, 3, 6, 9, 12 and 24h after treatment. Values were means of three replicates. Significant differences were denoted by different letters, *P* < 0.05, Student t test

Under normal growth conditions*,* the *GhABF3* exhibited constitutive expression pattern in different organs, with relatively higher transcript abundance in root, leaf, sepal, ovule and stamen, but lower level in stem (Fig. 2a). We analyzed the expression pattern of *GhABF3* via qRT-PCR in cotton under abiotic stress. Results indicated that upon ABA treatment, *GhABF3* expression in leaf increased and reached a four times peak that of the control at 6h (Fig. 2b). Under salt stress, the expression level of *GhABF3* in root tissue increased to 3.69-fold at 9 h, and then slowly decreased to normal level (Fig. 2c). In addition, after PEG6000 treatment, *GhABF3* expression in root gradually increased until reaching a peak at 12h after treatment (Fig. 2c). Overall, the above results indicated that *GhABF3* was induced and upregulated by salt, drought and exogenous ABA treatment, and involved in the response of cotton to salt and drought stress; and ABA treatment.

### Silencing of *GhABF3* through VIGS decreased salt and drought tolerance in cotton

**Fig. 3:**
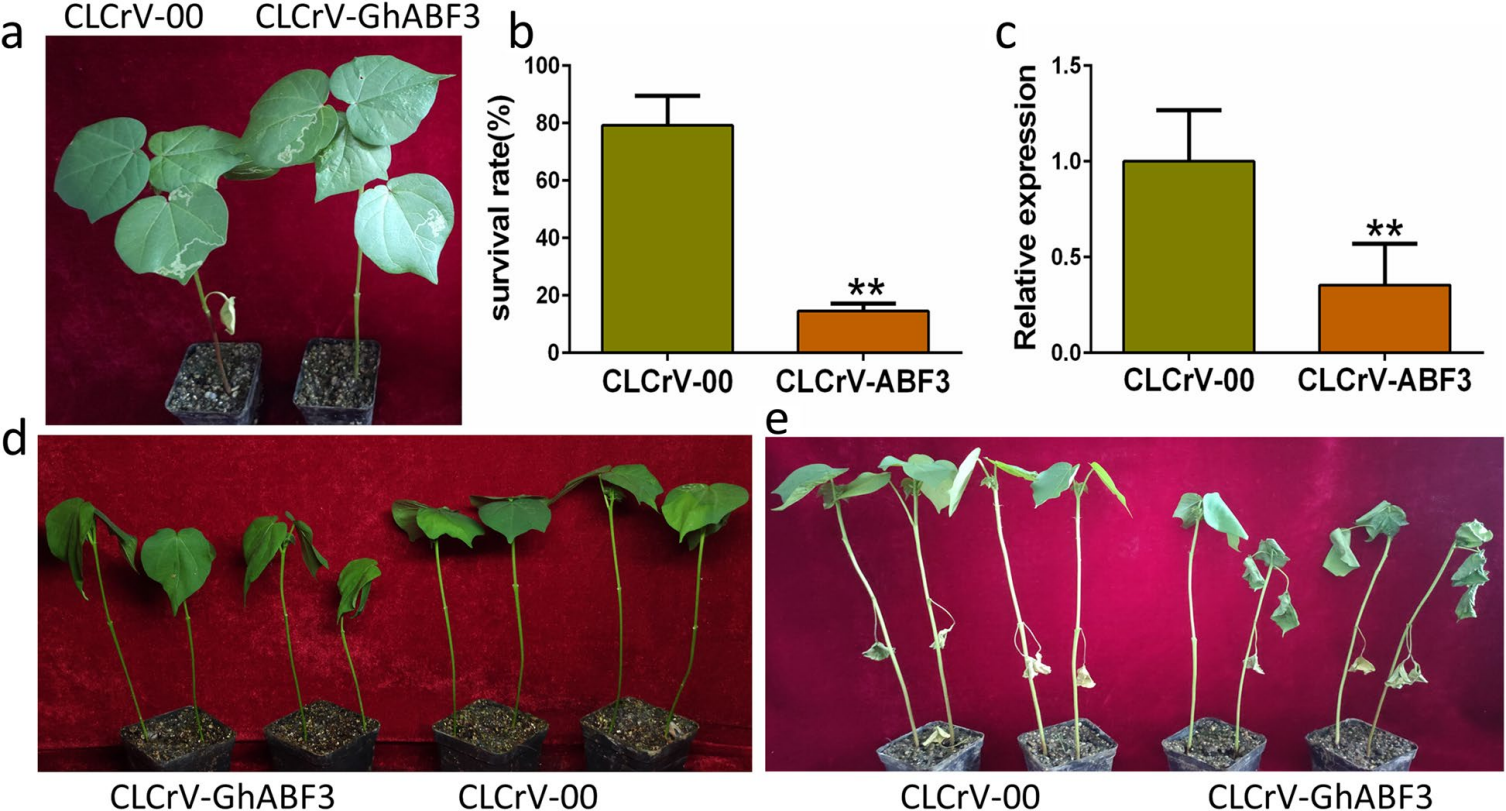
Silencing of GhABF3 resulted in reduced salinity and drought tolerance in cotton. **a** Phenotype of untreated control plants. **b** The survival rates were calculated after re-watering for 2 days. (**c**) Detection of silencing efficiency against CLCrV-GhABF3 plants after positive control appeared albino phenotype. **d**,**c** CLCrV-GhABF3 cottons exhibited more sensitivity to salt **d** and drought **e** stress than CLCrV-00 plants. Values were means of three replicates. **P ≤ 0.01; Student t test

**Fig. 4:**
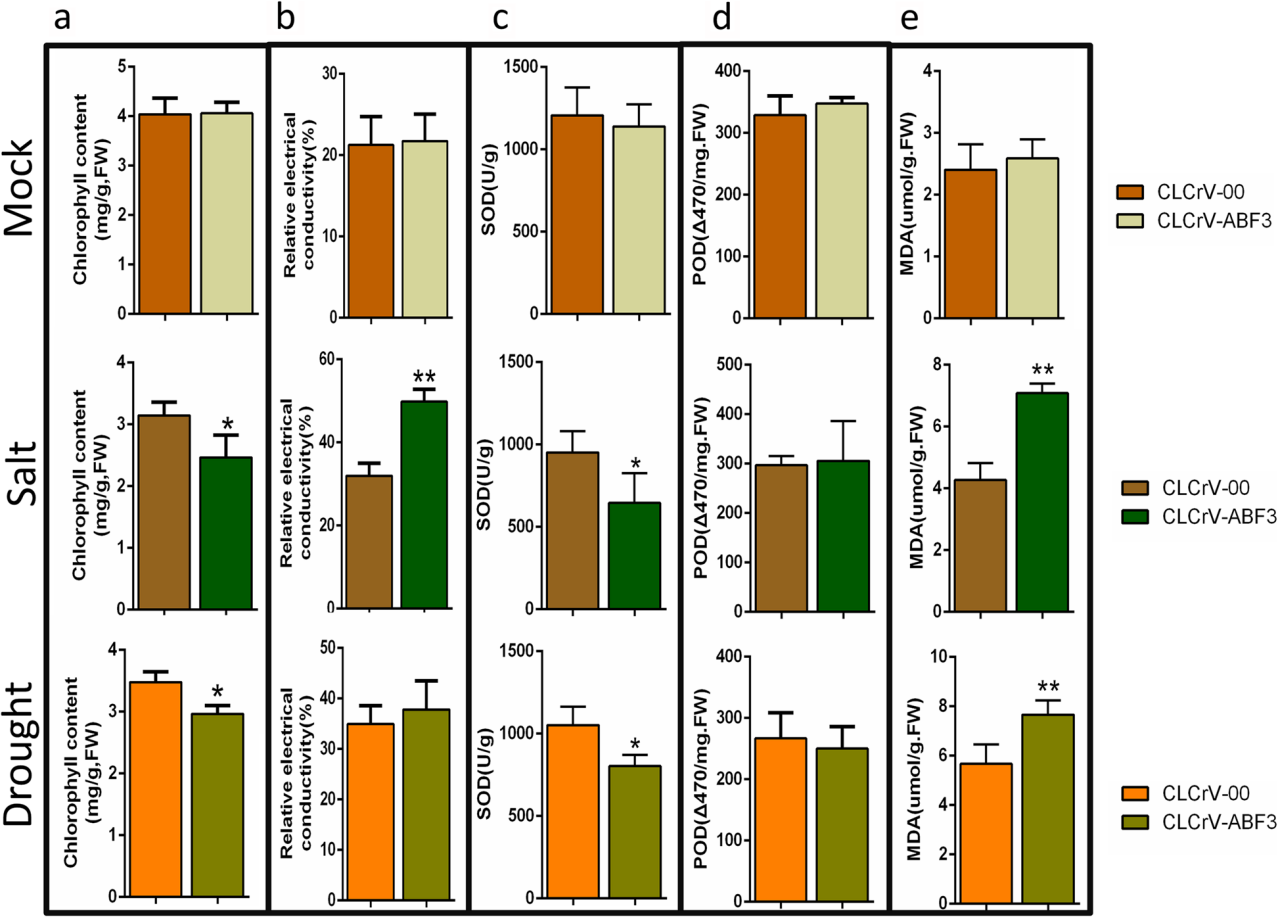
Drought and salt stress resulted in more severe cell damage in GhABF3 silenced plants. Changes in chlorophyll content (**a**), relative electrical conductivity (**b**), superoxide dismutase (**c**), peroxidase (**d**) and malondialdehyde (**e**) content in cotton leaves after water, drought and salt stress treatments. Values were means of three replicates.*P≤ 0.05, **P ≤ 0.01; Student t test

To initially validate the function of *GhABF3* in cotton, we used CLCrV-based virus-induced gene silencing (VIGS) to silence the gene. Two weeks after Agrobacterium tumefaciens infiltration, the transcript level of *GhABF3* was reduced by 64.54% in CLCrV-GhABF3 plants, indicating that *GhABF3* was efficiently silenced (Fig. 3c). Under normal growth conditions, the silenced plants showed no obvious difference in growth characters compared to empty vector control plants (Fig. 3a). After 15 days of 400mM NaCl stress, compared with the control, CLCrV-GhABF3 cotton exhibited more severe leaf wilting (Fig. 3d), accompanied by significantly increased leaf relative electrical conductivity (REC) and malondialdehyde (MDA) content (Fig. 4b, e). In addition, chlorophyll content (2.46mg/g) and superoxide dismutase (SOD) activity (646.16U/g) were significantly lower in the CLCrV-GhABF3 cotton leaves than in the CLCrV-00 plants (3.14mg/g, 951.69U/g) (Fig. 4a, c).

The drought stress tolerance of CLCrV-GhABF3 cotton was also evaluated. Results indicated that both *GhABF3* silenced plants and control plants under two-week none irrigation showed serious wilting symptoms and cotyledon shedding. There were no significant differences between the silenced and the control plants. But significant biochemical changes were detected between the two groups of plants after drought stress. The chlorophyll content (2.96mg/g) and SOD activity (802.82U/g) of leaves in the *GhABF3* silenced group were significantly lower than those in the CLCrV-00 group (3.45mg/g, 1051.69U/g) (Fig. 4a). The MDA content in leaves of CLCrV-GhABF3 plants (7.63 umol/g) was significantly higher than that of CLCrV-00 plants (5.92 umol/g) (Fig. 4e). However, there was no significant difference in REC and peroxidase (POD) activity between the two groups of plants (Fig. 4b, d). Furthermore, after two days of re-watering, the CLCrV-00 plants gradually recovered growing at a survival rate of 79.22%, which was significantly higher than 14.55% of the *GhABF3* silenced group (Fig. 3b). This indicated that most CLCrV-GhABF3 plants were irreversibly damaged, resulting in plant death (Fig. 3e). It could be seen from the results that drought before re-watering had similar damage to both cells, but the control group could slowly recover normal growth through re-watering, while the plants could not recover normal growth after *GhABF3* was silenced.

### Overexpression of *GhABF3* enhances abiotic stress tolerance of *Arabidopsis thaliana*

**Fig. 5:**
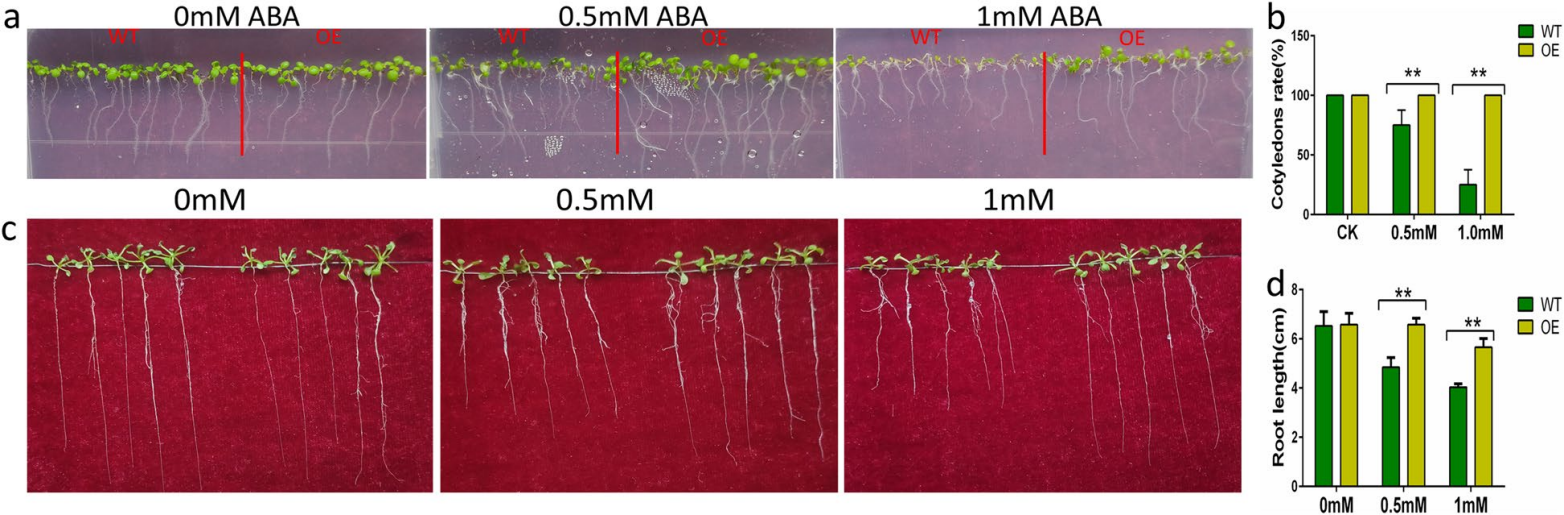
Overexpression of GhABF3 reduces the sensitivity of Arabidopsis to ABA. (a) Phenotype of Arabidopsis after 7 days of culture in medium containing 0, 0.5 and 1mM ABA, (b) Green leaf rate of plants after ABA treatment for 7 days. (c) Phenotype of Arabidopsis after 14 days of culture in medium containing 0, 0.5 and 1mM ABA. (d) Root length comparison after ABA treatment for 14 days. Each data value was from three replicated experiments. Significant differences were denoted by **, meaning P < 0.01 (Student T-test).

To further verify the gene function of *GhABF3*, we compared difference in response to drought, salt and ABA treatment between gene overexpression (OE) and col-0 (WT) lines of Arabidopsis. After 7 days of culture in 1/2 MS medium containing 0.5mM and 1.0mM ABA, the OE exhibited significantly larger cotyledons, longer root length and higher cotyledon extension rate than WT, and such differences became more obvious with the increase of ABA concentration (Fig. 5a, b). No obvious differences were observed between OE and WT plants cultivated without ABA treatment (Fig. 5a). After 20 days of treatment, the growth was consistent among OE and WT plants in the ABA-free culture dishes, while the root length of OE plants was significantly longer than those of WT under ABA stress (Fig. 5c, d).

**Fig. 6:**
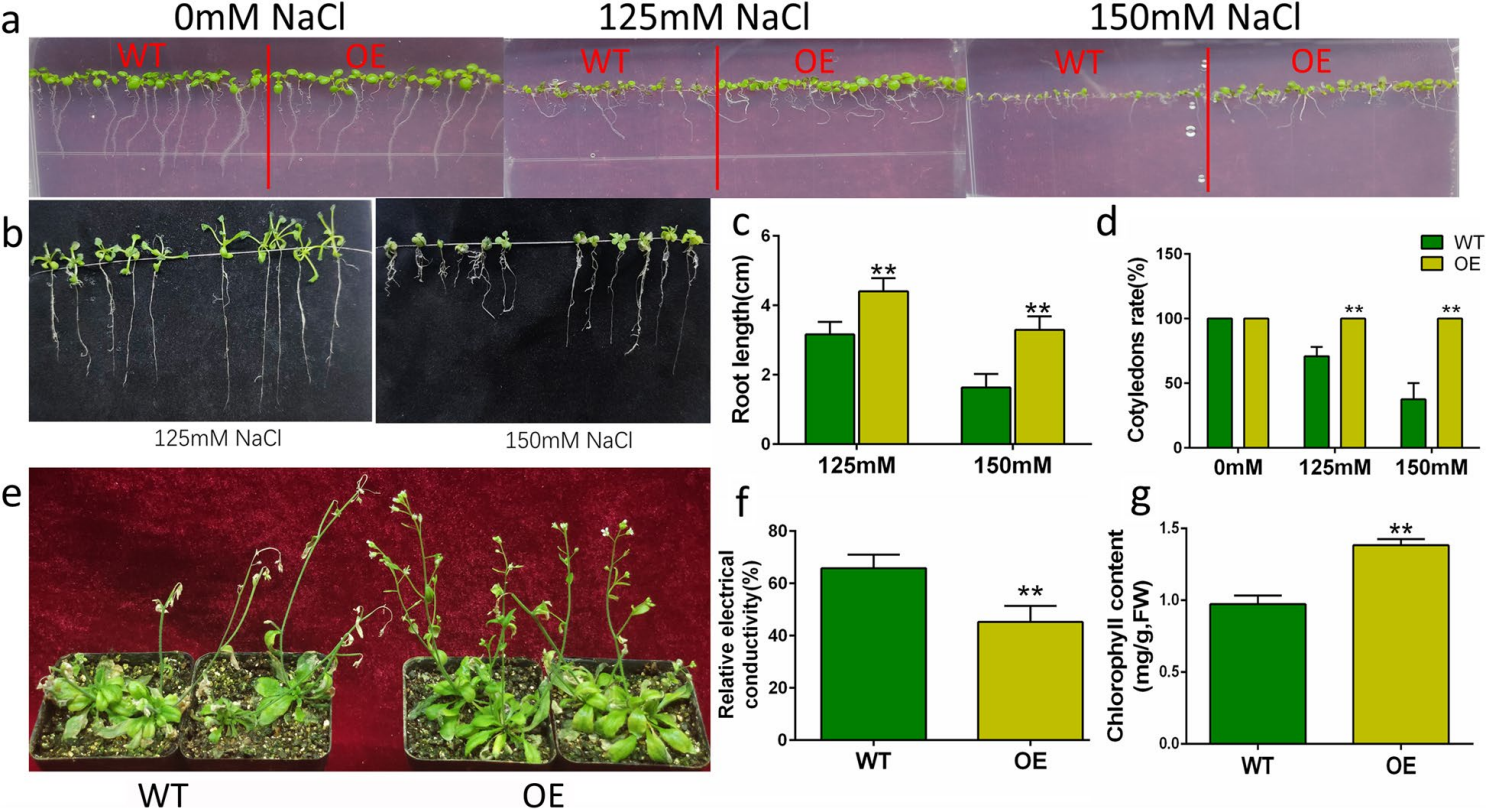
Overexpression of GhABF3 enhanced salt tolerance in Arabidopsis thaliana. Phenotypes of WT and OE plants grown in medium containing 0, 125, 150mM NaCl for 7 days (**a**) and 20 days (**b**). **c** Comparison of root length of WT and OE grown in medium containing 125 and 150 mM NaCl for 20 days. **d** Green leaf rate of WT and OE plants treated with salt at 7 days. **e** Phenotypes of WT and OE plants subjected to 200 mM NaCl stress for 7 days. Relative electrical conductivity (**f**) and chlorophyll content (**g**) of Arabidopsis leaves after 200mM NaCl treatment for 7 days. Each data value was from three replicated experiments. Significant differences were denoted by **, meaning *P* < 0.01 (Student T-test)

After 7 days of growth under normal conditions, there was no significant difference in the percentage of green leaves and root length between OE and WT lines (Fig. 6a). Under 125mM and 150mM NaCl stress, the green leaf rates of OE were significantly higher than those of WT, with increases of 30% and 59%, respectively (Fig. 6d). After 20 days of 150mM NaCl treatments, the root length of OE plants was twice as long as the WT (Fig. 6b, c).

The salt tolerance to more severe 200mM NaCl stress in OE Arabidopsis at adult-plant stage was further investigated. It was found that the leaves of wild-type rosettes turned yellow and died, and the flower stems withered. In OE plants, only a small portion of rosette leaves withered and died, while most of the leaves and flower stems grew normally and pods were produced (Fig. 6e). The REC of WT (65.83%) was much higher than that of OE plants (45.22%) (Fig. 6f), implying more serious damage in membrane system of WT than OE. The total chlorophyll content of the OE was 1.38mg/g, and that of the WT was only 0.97mg/g (Fig. 6g). These results suggested that exogenous overexpression of *GhABF3* improved salt tolerance of Arabidopsis during the whole growth period.

**Fig. 7:**
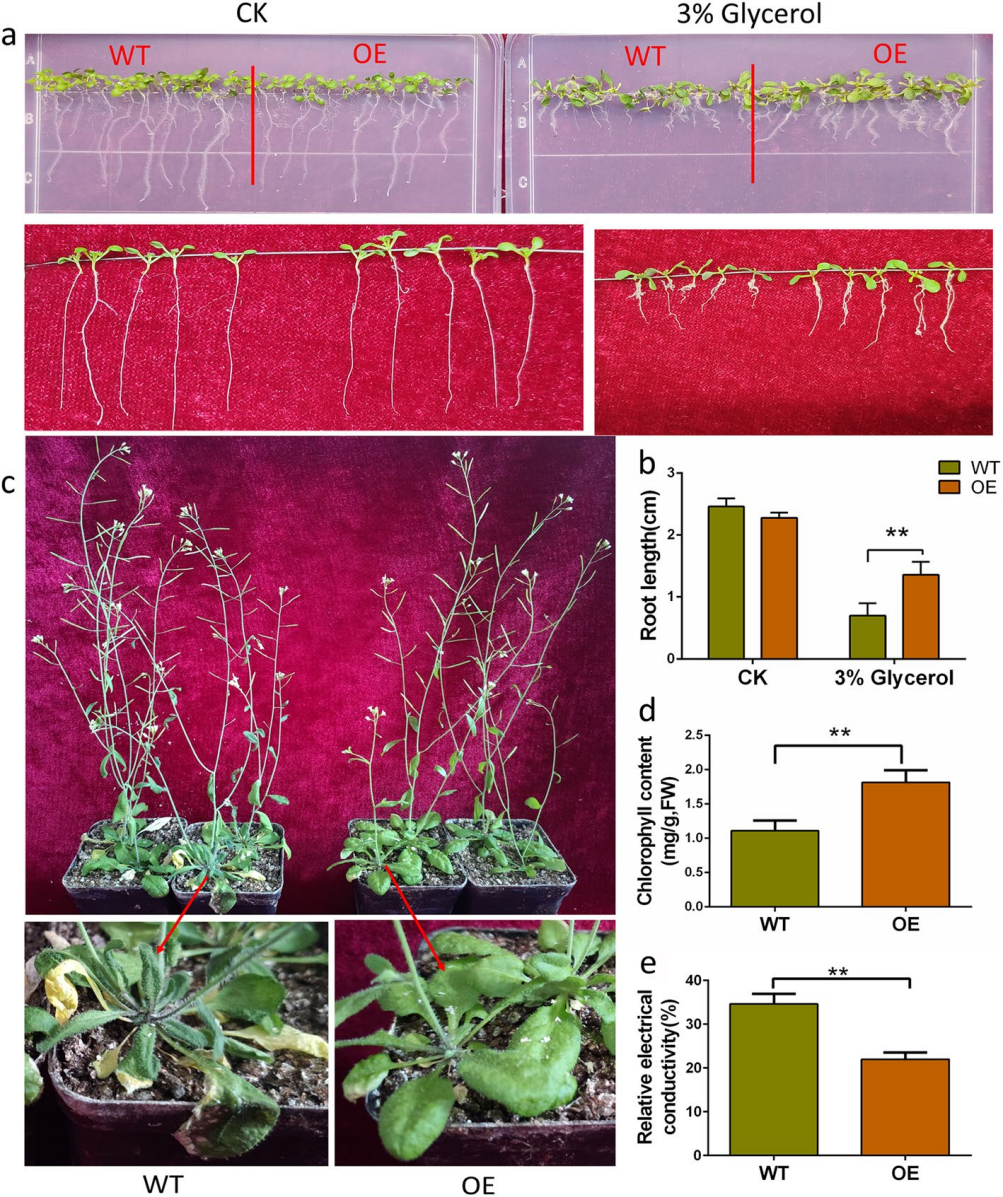
Overexpression of GhABF3 improves drought resistance in Arabidopsis. **a** Identification of phenotypes for drought resistance at seedling stage in Arabidopsis overexpressing GhABF3, 10 days under laboratory conditions **b** Root length comparison after 10 days of 3% glycerol culture. **c** Phenotypes of 30-day-old Arabidopsis after 7 days of natural drought. **d** Total chlorophyll content in leaves after 48 hours of re-watering. **e** Leaf relative electrical conductivity after 48 hours of re-watering. Each data value was from three replicated experiments. Significant differences were denoted by **, meaning *P* < 0.01 (Student T-test)

Drought was simulated with 3% glycerol in 1/2MS medium to evaluate drought resistance of Arabidopsis at seedling stage. After 10 days of growth without glycerol, there was no significant difference in the growth status of WT and OE lines (Fig. 6a). Under 3% glycerol stress, the root length of OE was about twice that of WT (Fig. 7a, b). After 7 days of natural drought at adult-plant stage, the OE leaves were relatively plump, while those of WT wilted and lose water seriously (Fig. 7c). The total chlorophyll content in leaves of *GhABF3* transgenic plants after re-watering was 1.81 mg/g, which was significantly higher than that of wild-type plants (1.11 mg/g) (Fig. 7d). The REC in wild-type plants (34.62%) was much higher than that in transgenic plants (21.98%) ((Fig. 7e), indicating that drought stress resulted in more severe damage in cells of wild-type plants. Based on the above results, it was suggested that exogenous overexpression of *GhABF3* could improve drought resistance of Arabidopsis at both seedling and adult-plant stages.

### Overexpression of *GhABF3* enhanced the stress resistance of cotton

**Fig. 8:**
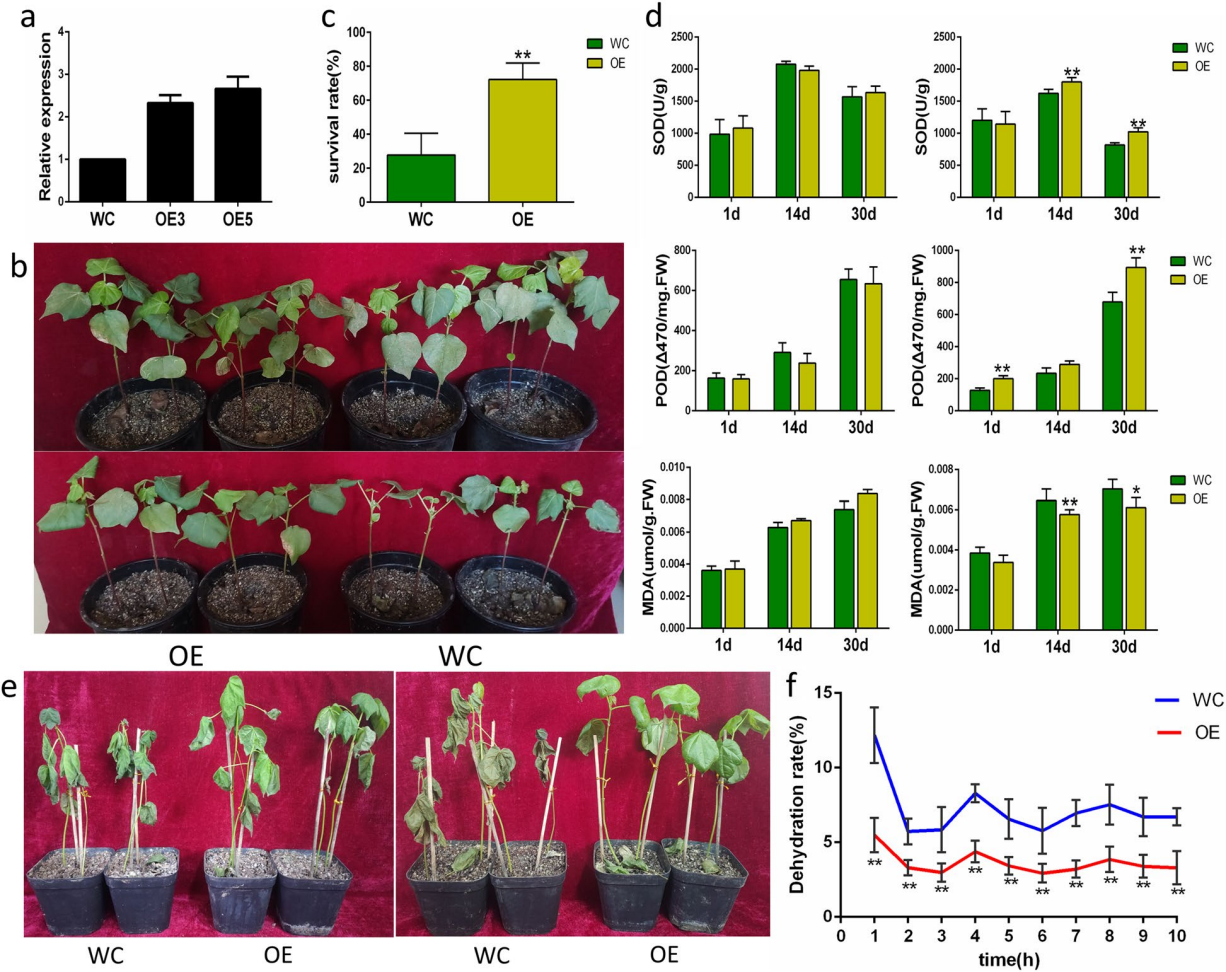
Overexpression of GhABF3 improves the drought and salt tolerance of cotton. **a** Identification of GhABF3 expression level in overexpressed plants. **b** Phenotype of cotton after salt treatment for 30 days. The upper part was the water treatment group; the lower part was the 300mM NaCl treatment group. **c** Survival rate after re-watering for 48h. **d** Changes of SOD, POD and MDA after treatment. The left was the water treatment group; the right was the salt treatment group. **e** Cotton phenotype under drought treatment, the left was after 2 weeks of natural drought, and the right was after 2 days of re-watering. **f** Dehydration rate of isolated leaves. Each data value was from three replicated experiments. Significant differences were denoted by * meaning *P* < 0.05; **meaning *P* < 0.01 (Student T-test)

To further confirm the *GhABF3* function in response to abiotic stresses in cotton, we developed transgenic cotton plants carrying a *35S::GhABF3* construct. A transgenic line OE5 having 2.66-fold higher transcript level as compared to wild type was chosen for further studies (Fig. 8a). Materials were subjected to 300mM NaCl irrigation and natural drought, respectively. After exposure to 300mM NaCl stress for 30 days, the number of healthy leaves of OE5 plants was significantly more than that of wild-type plants, and the growth points were healthier than those of wild-type plants (Fig. 8b). After natural drought for two weeks, the leaves of OE5 cotton were significantly less wilted than those of WC (*G. hirsutum*) variety plants (Fig. 8e).

The activity of some stress tolerance related enzymes SOD, POD and MDA was investigated in OE5 and WC variety upon salt stress. It was found that in the water treatment group, with the increase of leaf age, SOD activity first increased and then decreased, while POD activity and MDA content continued to increase. However, these indicators in the water treatment group were not significantly different between OE5 and WC variety plants (Fig. 8d). In the leaves of OE5 plants, the activity of SOD was significantly higher than that of WC variety plants after 14d and 30d of salt treatment. The activity of POD was significantly higher than that of WC variety plants after 1d and 30d of salt treatment, while the content of MDA was significantly lower than those of WC variety plants after 14d and 30d of salt treatment (Fig. 8d). The above results indicated that compared with the wild type, overexpression of *GhABF3* significantly improved the antioxidant capacity of cotton under stress, improved the ability to remove peroxides, and reduced the accumulation of harmful substances to protect the normal growth of cells.

On re-watering after drought treatment, the survival rate was counted after two days. It was found that the survival rate of OE5 plant was 72.2%, significantly higher than that of WC variety cotton (27.8%) (Fig. 8c). In addition, the in vitro leaf dehydration rate of WC variety and OE5 cotton leaves were also examined (Fig. 8f). During the whole dehydration process, the dehydration rate of OE5 was significantly lower than that of WC variety. The biggest difference between the two groups appeared at the beginning of dehydration, after which the dehydration rate of WC variety decreased rapidly but remained at significantly higher level compared with that of OE5. These results indicated that overexpression of *GhABF3* could improve the leaf water retention ability, and thus improved the drought resistance of cotton plants.

### Overexpression of *GhABF3* regulates the expression of genes involved in stress response

**Fig. 9:**
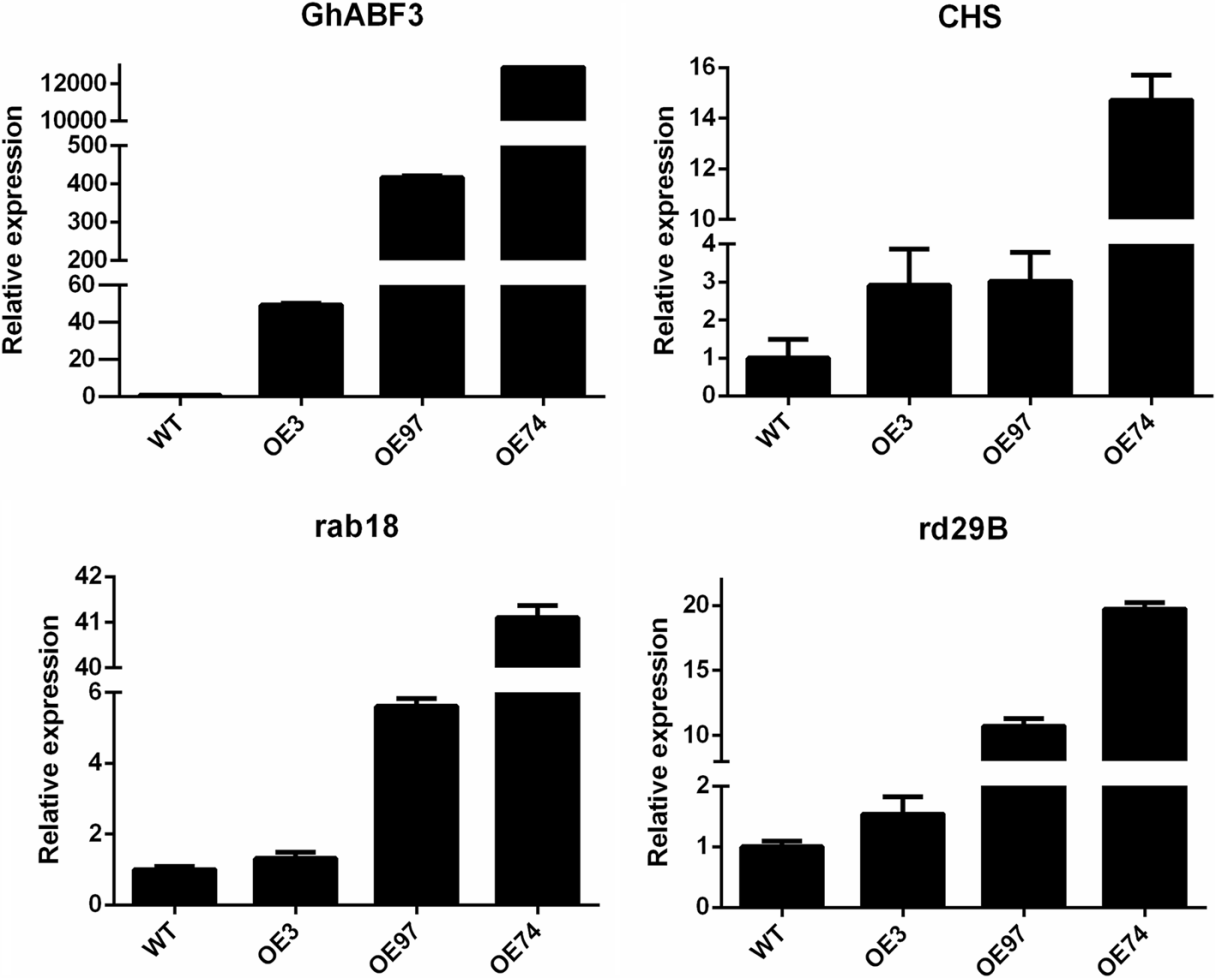
Expression analysis of stress response genes in GhABF3 transgenic lines and wild-type Arabidopsis. The relative expression of all genes was analyzed by qRT-PCR, and the expression level of WT plants was defined as 1 under normal growth conditions. Three independent experiments were conducted, each of which involved at least three plants

To further study the mechanism by which *GhABF3* regulates the downstream stress-related genes at the transcriptional level, the expression of three stress/ABA-responsive genes were measured by qPCR in three transgenic Arabidopsis lines (OE3, OE74 and OE97). The expression level of *GhABF3* gene in OE3, OE74 and OE97 was 49, 417, and 12900 times that of wild-type, respectively. It was found that *rd29B*, *rad18* and *CHS* genes were all up-regulated in three OE lines under salt stress, and the expression levels increased with the increase of *GhABF3* gene expression (Fig. 9). These results indicated that *rd29B*, *rad18* and *CHS* could respond to the regulation of *GhABF3* gene. Our results suggested that *GhABF3* might enhance stress resistance by increasing downstream stress/ABA-related gene expression.

## Discussion

ABA is an important phytohormone that controls responses to abiotic stresses [30]. Studies have shown that ABA is involved in plant response to various stresses, such as drought, low temperature, high temperature, salt, etc. [31]. A series of terminal transcription factors, such as ABFs, regulate plant responses to abiotic stress by regulating downstream ABA-inducible genes [32]. Some downstream ABA-inducible genes have been identified in Arabidopsis, such as *rd29A*, *rd29B*, *CHS*, *rab18 *[33–35]. In this study, we isolated and identified *GhABF3* with typical bZIP domain BRLZ from upland cotton, and found that salt treatment up-regulated the expression of *rd29B*, *CHS* and *rab18* in *GhABF3* transgenic Arabidopsis. Under the influence of exogenous ABA, the cotyledon growth and root growth of transgenic Arabidopsis seedlings were significantly better than those of wild type. These results suggested that *GhABF3* was involved in plant responses to stress via an ABA-dependent manner.

When faced with abiotic stresses, plants can enhance their resistance through complex signal-regulatory systems in the body. Among them, AREB/ABF acts as a transcription factor at the end of the ABA signaling pathway in response to abiotic stresses. Nine AREB/ABF transcription factors were previously identified in Arabidopsis [32]. *AtABF3* was up-regulated in Arabidopsis vegetative tissues under ABA, drought and salt treatments [36–38], and the expression pattern of *GhABF3* in upland cotton in this study was similar to that in Arabidopsis. The timing of *ABF3* response to ABA stress varied in different crops. The *DcABF3* expression was highest at 12 h after ABA treatment in carrot [26], whereas *AtABF3* was upregulated by ABA treatment and the expression level was highest 2 h after treatment in Arabidopsis [39]. Here, the *GhABF3* expression reached its highest level in cotton leaf at 6 h post ABA stress. In cotton, another *ABF* (*GhABF2*) was previously reported to be down-regulated by salt stress. These results suggested that ABFs were involved in the response of plants to abiotic stress, while the mechanism of action of specific genes was different in different crops and different members of the ABF family.

Plants absorb water and nutrients mainly through their roots, so strong root system can provide more water and nutrients to plants [40]. Salting is toxic to plants and inhibits plant growth, including reducing photosynthetic rate; and inhibiting root and stem elongation [41]. Exorbitant soil salt content also leads to osmotic stress, making plant roots absorbing water difficult [42, 43]. Under salinity and drought stress, Arabidopsis heterologously expressing *GhABF3* had longer roots than the wild-type, enabling them to explore and extract water and nutrients from the soil. Normal chlorophyll content is essential for maintaining leaf function and photosynthesis rate, while chlorophyll deficiency under stress conditions will lead to leaf senescence [44, 45]. In this study, through the dual identification in Arabidopsis and cotton, the *GhABF3* overexpression could delay leaf senescence under salt and drought stress by reducing chlorophyll degradation. Similar results has been previously reported in *AtABF3* transgenic alfalfa leaves [25]. Conversely, *AtABF3* mediated ABA-induced chlorophyll degradation in *A. thaliana*, and triple recessive abf2abf3abf4 significantly reduced the chlorophyll degradation rate after ABA induction [23]. Obviously, whether *ABF3* plays a consistent role in chlorophyll degradation under different stress treatments remains to be further explored.

Reactive oxygen species (ROS) are continuously produced in plants as by-products of aerobic metabolism. Under normal growth conditions, plants produce ROS as a signaling molecule to regulate different metabolic reactions [46]. When oxidative stress occurs, excessive accumulation of reactive oxygen species (O^2ˉ^ and H_2_O_2_) in plants can lead to cellular oxidative damage such as cell membrane leakage [47]. Relative electrical conductivity (REC) reflects the extravasation of electrolytes in plant cells and represents the degree of damage to plant cell membranes [48, 49]. MDA is a product of membrane lipid peroxidation, which can aggravate membrane damage [50, 51]. The present work indicated that under abiotic stress, overexpression of *GhABF3* reduced the leaf REC and MDA content in Arabidopsis and cotton, whereas silencing *GhABF3* by VIGS increased both MDA content and REC of cotton leaves. Plants can enhance the scavenging ability of ROS and reduce the damage of ROS by maintaining high antioxidant enzymes activity [52–54]. SOD protects cells from oxidative damage by dismutating superoxide anion radicals to generate hydrogen peroxide and molecular oxygen [55]. POD is a kind of oxidoreductase and has the dual function of eliminating the toxicity of hydrogen peroxide and phenolamine [56]. This experiment showed that SOD and POD activities were positively correlated with the overexpression of *GhABF3* in cotton under stress, whereas in *GhABF3* silenced cotton under stress, only SOD activity decreased significantly with little reduction in POD activity. Similar SOD activity change was also detected in cotton overexpressing *GhABF2* upon salt stress [21]. Notably, such changes in antioxidant enzyme activity occurred only under stress, while there was no significant change in the activity of these enzymes under normal growth conditions, whether in *GhABF3* overexpression or silenced cotton plants.

## Conclusions

In this research, the *GhABF3* gene was successfully cloned from upland cotton, and its expression was induced by drought, salt and ABA. Overexpression of *GhABF3* in cotton and *A. thaliana* exposed to abiotic stress improved multiple resistance characteristics, including increases in root length, SOD and POD activity, and reduction in chlorophyll degradation and cell damage. Silencing of *GhABF3* results in reduced resistance to abiotic stress in cotton. Taken together, *GhABF3* responded to abiotic stress through an ABA-dependent way, and manipulation of *GhABF3* expression could enhance plant resistance to salt and drought stress. The results will facilitate the breeding program of resistant cotton cultivars.

## Materials and Methods

### Selection and naming of *GhABF3*

In our laboratory, a large number of genes responding to salt stress were found in the transcriptome profiling of the *G. hirsutum* cultivar Han682 at the germination stage under salt stress. *Gh_D12G0214* was one of the genes responding to salt stress. The homologous gene *Gh_A12G0212* on the A subgenome had 95.63% amino acid sequence (Fig. S3) and 96.25% base sequence (Fig. S4) similarity with *Gh_D12G0214*. However, the expression of *Gh_A12G0212* was low, so the *Gh_D12G0214* gene was selected for further functional characterization (Table S2).

*Gh_D12G0214* was named as *GhABF3* in Cotton Functional Genomics Database (CottonFGD) (https://cottonfgd.org/). Clustering analysis of this gene and the Arabidopsis ABF family genes revealed that this gene was the closest to *AtABF3* (Fig. S1) and had the highest amino acid sequence similarity (51.94%) with *AtABF3* (Fig. S2). Therefore, the nomenclature of *GhABF3* was used in this study.

### Plant material selection and growing conditions

The coding sequences of *GhABF3* were cloned from cDNA of Han682, which was developed by Handan Academy of Agricultural Sciences (Handan, China). WC is a *G. hirsutum* variety selected by the Cotton Research Institute of Shanxi Academy of Agricultural Sciences (Taiyuan, China) [57], was selected as the transgenic cotton recipient genotype. And the transgenic Arabidopsis recipient genotype was Colombia wild type (Col-0). The WC variety cotton seeds were donated by Prof. Shenjie Wu from Shanxi Agricultural University. The cotton growing environment under laboratory conditions was keep in 16h light (25℃) / 8h dark (25℃). Arabidopsis was planted in 1/2 MS culture medium, at 4℃ cooling off two days later transferred to the incubator. When Arabidopsis grew to two true leaves, it was transplanted into the soil for culture, all the time 16h light (23℃) / 8h dark (23℃) light cycle was kept. All plant material sampled for research in this experiment required no permissions.

### Stress treatment for gene expression analysis

Han 682 cotton plants, grown in soil in a growth chamber for 4 weeks, were used for the *GhABF3* qRT-PCR relative expression assays. Every two cotton seedlings in the same growth state were taken as a biological replicate. Water treatment was taken as the control group. For exogenous ABA treatment, leaves were sprayed with 500μM ABA solution. For salt stress test, 250mM NaCl solution was poured until the soil was saturated. For the water deficit assay, 10% PEG6000 solution was soaked to simulate drought stress. Seedlings were sampled at 0, 3, 6, 9, 12, and 24h after treatment. RNA was isolated from leaves and roots, respectively. In order to detect the difference of *GhABF3* expression in different organs, we isolated roots, stems, leaves, petals, stamens, pistils, sepals, and ovules from 3-month-old cotton and performed RNA extraction.

### RNA extraction and qRT-PCR analysis

RNA was extracted from samples using the OminPlant RNA Kit (DNase I) (CWBIO, Beijing, China), and cDNA was prepared using the Hifiscript cDNA Synthesis Kit (CWBIO, Beijing, China). NCBI was used to design primer RTABF3 for qRT-PCR, *GhUBQ14* as internal control [58]. Gene expression levels were calculated using 2^-ΔΔCt^, where Ct was the cyclic threshold. Primers for PCR are listed in Table S1.

### Generation of transgenic plantss

*GhABF3* encoding region was amplified by the KOD DNA Polymerase (Toyobo, Shanghai, China) from Han 682 (Fig. S5b), and then ligated into the pCAMbia2300 plant overexpression vector using KpnI and BamHI enzymes to obtain the *35S::GhABF3* vector (Fig. S7, Fig. S11). Col-0 Arabidopsis thaliana was used as the recipient genotype. Transgenic Arabidopsis plants were obtained using the floral dip method [59]. The sterilized seeds of T_0_ generation were sown on 1/2 MS (containing 35mg/L Kana) screening medium, and the seedlings with young roots and green leaves were transplanted and cultivated. The leaf DNA of Arabidopsis thaliana seedlings after initial screening was extracted, and the *GhABF3* gene was amplified by PCR to obtain T_1_ generation positive transgenic Arabidopsis (Fig. S8a). Continued to use 1/2 MS (containing 35mg/L Kana) screening medium to purify and screen the T_2_ generation transgenic Arabidopsis that meet the 3:1 segregation patterns (Table S3). Until the homozygous T_3_ generation seeds were obtained, they were used for subsequent experiments.

The *GhABF3* transformation of WC variety was carried out in the field by pollen-tube transformation method [60]. The *35S::GhABF3* overexpression vector plasmid was extracted by alkali cracking method, and diluted to 500ng/ul. Plasmid injections were given between 6 a.m. and 8:30 a.m. the day after flowering. The T_0_ seedlings were firstly screened by 4000ppm kanamycin spraying, then positive plants were verified by *GhABF3* gene amplification using 35SF+ABF3KpnI primers, and further verified by PCR product sequencing (Fig. S8b). The T_2_ lines (OE3 and OE5) that meet the segregation patterns of 3:1 were continued to cultivate (Table S3), and obtained homozygous T_3_ generation seeds for subsequent experiments.

### VIGS of *GhABF3* in cotton and evaluation of stress resistance

*GhABF3* gene was silenced via Agrobacterium infiltration approach [61]. The pCLCrV-GhABF3 vector (Fig. S6, Fig. S10) was transformed into Agrobacterium tumefaciens strain LBA4404 to infect 7-day-old Han 682 cotyledons. The pCLCrV-00 was taken as a negative control and pCLCrV-CLA1 as a positive control [61]. When plants showed a positive albino phenotype, leaf samples were taken for qPCR using RTABF3 primers to detect silencing effect. Then *GhABF3* silenced plants were evaluated for abiotic stresses resistance. Salt stress treatment was performed using 400mM NaCl. The trays in which the pots were placed were kept with NaCl solution at all times so that the soil absorbed water from the bottom of the pots. After 15 days of salt stress treatment, the activities of SOD and POD and the content of MDA in the plant leaves were measured. For drought treatment, watering was withheld for 2 weeks after the soil absorbing water to saturation, and then re-watering. Then their survival rates were calculated two days after re-watering. In addition, leaf chlorophyll content and relative electrical conductivity were measured after drought and salt stress treatment, respectively. Experimental materials were planted in 7×7.5 cm plastic pots with 2 plants per pot. Each stress treatment consisted of three biological replicates with four seedlings per replicate. Each replicate had three technical replicates. PCR primers were shown in Table S1.

### Stress tolerance assay of transgenic Arabidopsis

Tolerance to three abiotic stresses, including drought, salt and ABA, were measured in *GhABF3* overexpression Arabidopsis lines (OE74) and Col-0(WT) plants at young seedling and adult stages, respectively. As for young seedling stage tests, materials were sown on demand in 1/2MS medium containing different stress factors, including three ABA levels of 0uM, 0.5uM and 1uM, three densities of NaCl of 0mM, 125mM and 150mM as salt stress, and two concentrations of 0% and 3% glycerol as drought stress, respectively.

To evaluate abiotic stress resistance at adult stage, pots planted with 40-day-old Arabidopsis (WT and OE) were soaked in an appropriate amount of 200mM NaCl solution for 7 days as salt stress; 30-day-old Arabidopsis plants (WT and OE) were irrigated to saturation, and then suffered to 7 days natural dry and followed re-watering for 24 hours as drought stress. After stress treatment, the chlorophyll content and relative electrical conductivity in fresh leaves were determined. Experimental materials were planted in 7×7.5 cm plastic pots with 2 plants per pot.

### Expression detection of ABA-inducible genes in *GhABF3* transgenic Arabidopsis

Three transgenic lines OE3, OE74 and OE97, which conformed to the 3:1 segregation pattern and had different expression levels of *GhABF3*, were selected for identify the expression levels of ABA-inducible genes. 4-week-old Arabidopsis (WT and OE) were irrigated with 150 mM NaCl for 9 hours, their leaves were taken for RNA extraction and reversed transcribed (same method as above). Then, qRT-PCR was performed using RTABF3 primers (Table S1) and *AtActin* gene was used as an internal control [62].

### Stress tolerance assay of *GhABF3* transgenic cotton

Four-week-old *GhABF3* overexpressed plants and WC variety plants were irrigated with 300mM NaCl until soil water saturation, and distilled water was used as control. Enzymes (SOD, POD and MDA) activity/concentration in leaves were measured at 1, 14, 30 days post stress treatment. For drought treatment, plants (transgenic cotton and WC variety) were withheld watering for 2 weeks after the soil absorbing water to saturation, and then their survival rate was calculated after 2 days of rewatering. In addition, the drought resistance of plants was evaluated by calculating the dehydration rate of isolated leaves.

### Determination of physiological and biochemical indicators

Dehydration rate of isolated leaves [15], total chlorophyll content [63], relative electrical conductivity [64], SOD, POD and MDA [65] determination methods were performed according to previous studies.

Four four-week-old cotton plants with the same growth status were selected, and counted as one biological replicate. The intact cotton leaves at the same position were cut with scissors and weighed on an electronic balance. Then the leaves were kept at room temperature and away from direct sunlight and wind, and weighed every 1 hour. Leaf dehydration rate = (W_i_-W_i+1_)/W_i_, W_i_ was the leaf weight at time point i and W_i+1_ was the leaf weight at time point i+1.

The 0.2g of fresh leaves were taken from four plants, added 3ml of 95% ethanol and ground thoroughly. After centrifugation, 0.5 mL supernatant was taken to a constant volume of 5mL. The absorbance values at 649nm and 665nm were determined with 95% ethanol as blank control. Chlorophyll a (mg/L) =13.95A_665_-6.88A_649_, and chlorophyll b (mg/L) =24.96A_649_-7.32A_665_. Total chlorophyll content = chlorophyll a + chlorophyll b.

The 0.1g leaves (from four plants) were cut into slices and added 10ml distilled water. The conductivity named C1 was measured after constant temperature and vibration at 25℃ for 1h. The sample was boiled for 15min and then cooled to room temperature to determine the conductivity named C2. Relative electrical conductivity =C1/C2×100%.

The 0.25g of fresh leaves was taken, added 6 ml of pH 7.8 phosphate buffer, and grounnd into a slurry in an ice bath. The homogenate was refrigerated and centrifuged for 20 min, and the supernatant was stored in a 0-4 °C refrigerator for later use. The activity of POD was determined by guaiacol colorimetric method, the content of SOD in plant tissues was determined by nitro-blue tetrazolium photoreduction method, and the content of MDA was determined by colorimetric method.

### Statistical analysis of data

For the accuracy of the experiment, biological repetition setting, data detection and measurement were repeated more than three times in this study. Statistical analyses were conducted with SPSS 20.0. The mean values and the standard deviations of repeats were presented, and significant differences were shown by Student’s t–test.

## Supplementary Information

**Additional file 1: Fig. S1** Phylogenetic analysis of GhABF3 and nine Arabidopsis ABF family genes. **Fig. S2** Amino acid sequence comparison of GhABF3 and AtABF3. The red line part is the domain. **Fig. S3** Comparison of GhABF3 amino acid sequences of G. hirsutum A genome (Gh_A12G0212) and D genome (Gh_D12G0214). **Fig. S4** Comparison of GhABF3 CDS sequences of G. hirsutum A genome (Gh_A12G0212) and D genome (Gh_D12G0214). **Fig. S5** GhABF3 gene sequence cloning. (a) Agarose gel imaging of VIGS sequence cloning. (b) Agarose gel imaging of CDS sequence cloning. In order to ensure the simplicity of the image, the gel image is appropriately cropped. The original image is attached to Figure S9. **Fig. S6** Sequencing results of ClCrV-GhABF3 vector construction. Fig. S7 Sequencing results of 35S::GhABF3 vector construction. **Fig. S8** PCR identification of transgene positive transformation events. (a) PCR detection of GhABF3 transgenic Arabidopsis. (b) PCR detection of GhABF3 transgenic cotton. P stands for positive control, the substrate is the 35S::GhABF3 plasmid, N stands for negative control, and the substrate is the DNA of the transformed recipient plant. In order to ensure the simplicity of the image, the gel image is appropriately cropped. The original image is attached to Figure S9. **Fig. S9** Images of the original untreated gel used in Figures S5 and S8. (a) The original image used for Figure S5a. (b) The original image used in Figure S5b. On the left of marker is the used part in Figure S5b, and on the right is the band generated by other genes cloned at the same time. (c) The original image used in Figure S8a. (d) The original image used in Figure S8b. On the right of marker is the clipping part in Figure S8b, and on the left is the band generated by other genes simultaneously identified. **Fig. S10** The map of pCLCrV-GhABF3 vector. The arrow represents the direction of the insertion sequence. For more detailed carrier atlas information, please refer to [61] mentioned by predecessors. **Fig. S11** The map of 35S::GhABF3 vector. The sequence of the green arrow inside the circle is the inserted sequence, and the direction of the arrow represents the direction of the inserted sequence.

**Addtional file 2: Table S1.** Primer sequence. **Table S2.** Expression of *GhABF3* in Han682 germination salt-tolerant transcriptome sequencing.  **Table S3. **Transgene inheritance pattern of T_2_ plants. The expected ratio in the chi-square test was 3:1, χ^2^_0.05(1)_=3.84.

## Data Availability

The related gene sequence files of *G. hirsutum* (ZJU, TM-1) were downloaded from the CottonFGD (https://cottonfgd.org/). The Arabidopsis related gene sequence files are from the TAIR database (https://www.arabidopsis.org/). All data generated or analyzed during this study are included in this published article and its supplementary information files. The datasets used and analyzed during the current study are available from the corresponding author on reasonable request.

## Abbreviations

ABA: Abscisic acid
ABFs: Abscisic acid-responsive transcription factors
ABRE: ABA response element
AREB: ABA responsive element binding protein
CDS: Coding sequences
MDA: Malondialdehyde
POD: Peroxidase
PP2C: Protein phosphatase 2C
ROS: Reactive oxygen species
SnRK2: Sucrose non-fermenting 1-related protein kinase 2
SOD: Superoxide dismutase
TFs: Transcription factors
VIGS: Virus-induced gene silencing
