# Supplementary material for: Overexpression of GhABF3 increases cotton(Gossypium hirsutum L.) tolerance to salt and drought
Source: BMC Plant Biol. 2022 Jun 29;22:313. doi: 10.1186/s12870-022-03705-7 (PMC9241229; doi:10.1186/s12870-022-03705-7)
Supplement: Supplementary file 1 — Additional file 1: Fig. S1 Phylogenetic analysis of GhABF3 and nine Arabidopsis ABF family genes. Fig. S2 Amino acid sequence comparison of GhABF3 and AtABF3. The red line part is the domain. Fig. S3 Comparison of GhABF3 amino acid sequences of G. hirsutum A genome (Gh_A12G0212) and D genome (Gh_D12G0214). Fig. S4 Comparison of GhABF3 CDS sequences of G. hirsutum A genome (Gh_A12G0212) and D genome (Gh_D12G0214). Fig. S5 GhABF3 gene sequence cloning. (a) Agarose gel imaging of VIGS sequence cloning. (b) Agarose gel imaging of CDS sequence cloning. In order to ensure the simplicity of the image, the gel image is appropriately cropped. The original image is attached to Figure S9. Fig. S6 Sequencing results of ClCrV-GhABF3 vector construction. Fig. S7 Sequencing results of 35S::GhABF3 vector construction. Fig. S8 PCR identification of transgene positive transformation events. (a) PCR detection of GhABF3 transgenic Arabidopsis. (b) PCR detection of GhABF3 transgenic cotton. P stands for positive control, the substrate is the 35S::GhABF3 plasmid, N stands for negative control, and the substrate is the DNA of the transformed recipient plant. In order to ensure the simplicity of the image, the gel image is appropriately cropped. The original image is attached to Figure S9. Fig. S9 Images of the original untreated gel used in Figures S5 and S8. (a) The original image used for Figure S5a. (b) The original image used in Figure S5b. On the left of marker is the used part in Figure S5b, and on the right is the band generated by other genes cloned at the same time. (c) The original image used in Figure S8a. (d) The original image used in Figure S8b. On the right of marker is the clipping part in Figure S8b, and on the left is the band generated by other genes simultaneously identified. Fig. S10 The map of pCLCrV-GhABF3 vector. The arrow represents the direction of the insertion sequence. For more detailed carrier atlas information, please refer to [61] mentioned by predecessors. [file 12870_2022_3705_MOESM1_ESM.doc]

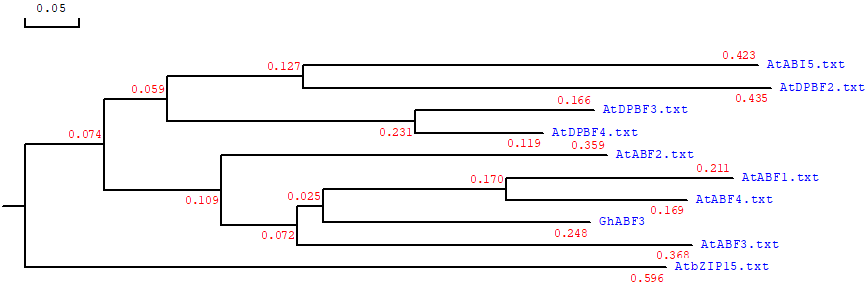


Fig. S1 Phylogenetic analysis of GhABF3 and nine Arabidopsis ABF family genes.


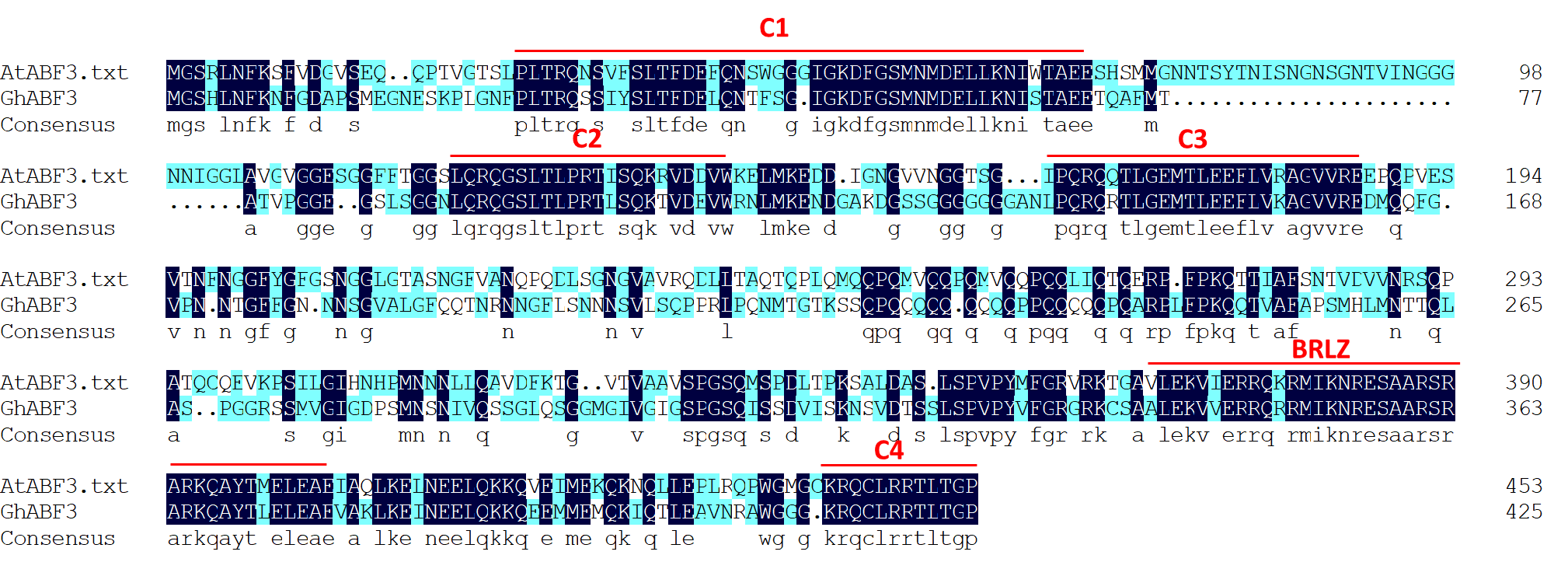


Fig. S2 Amino acid sequence comparison of *GhABF3* and *AtABF3*. The red line part is the domain.


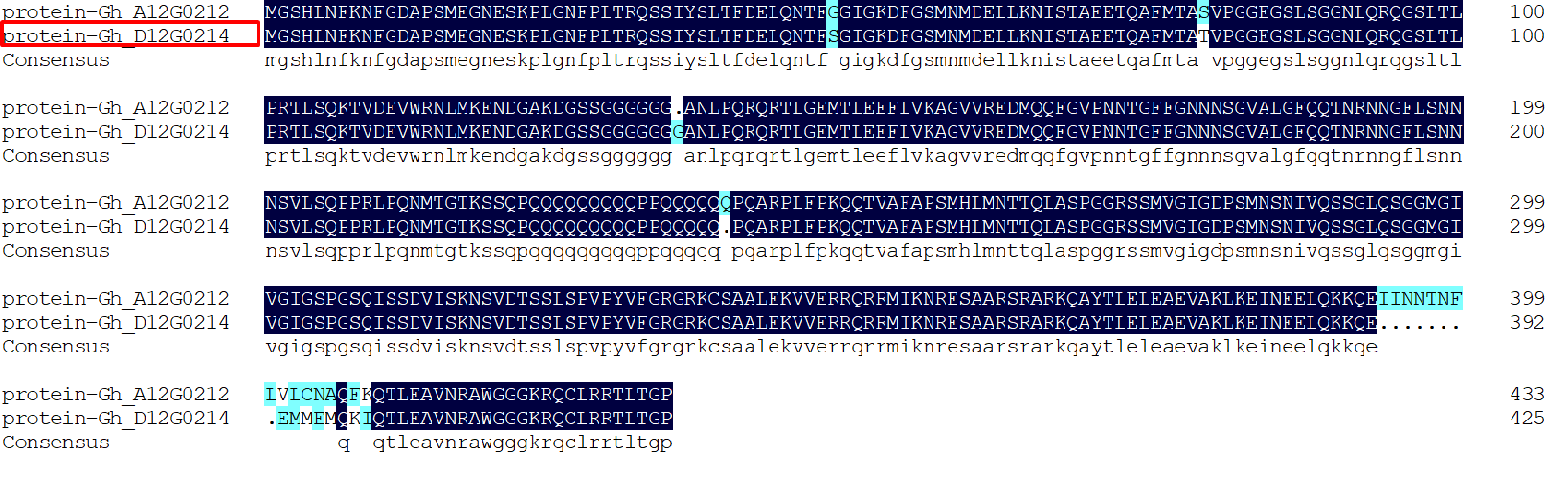


Fig. S3 Comparison of GhABF3 amino acid sequences of *G. hirsutum* A genome (Gh_A12G0212) and D genome (Gh_D12G0214).
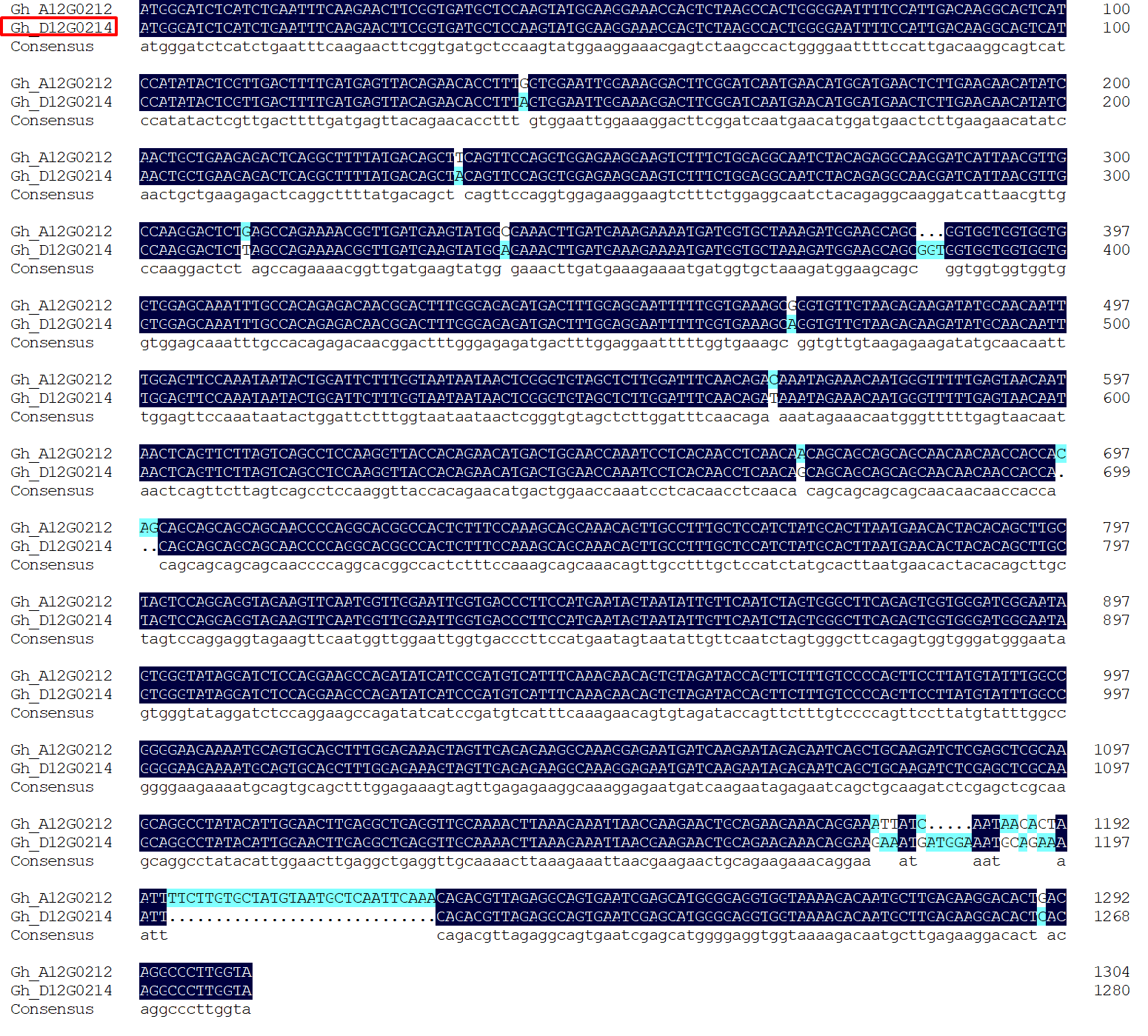


Fig. S4 Comparison of *GhABF3* CDS sequences of *G. hirsutum* A genome (Gh_A12G0212) and D genome (Gh_D12G0214).
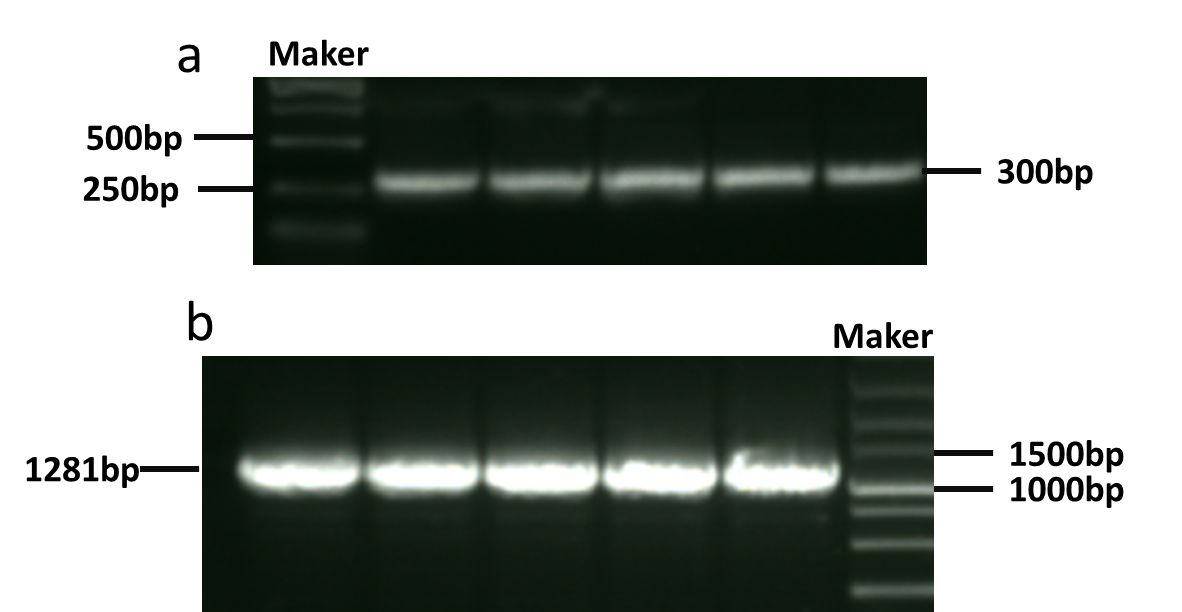


Fig. S5 *GhABF3* gene sequence cloning. (a) Agarose gel imaging of VIGS sequence cloning. (b) Agarose gel imaging of CDS sequence cloning. In order to ensure the simplicity of the image, the gel image is appropriately cropped. The original image is attached to Figure S9.

Fig. S6 Sequencing results of ClCrV-GhABF3 vector construction.

Fig. S7 Sequencing results of 35S::GhABF3 vector construction.


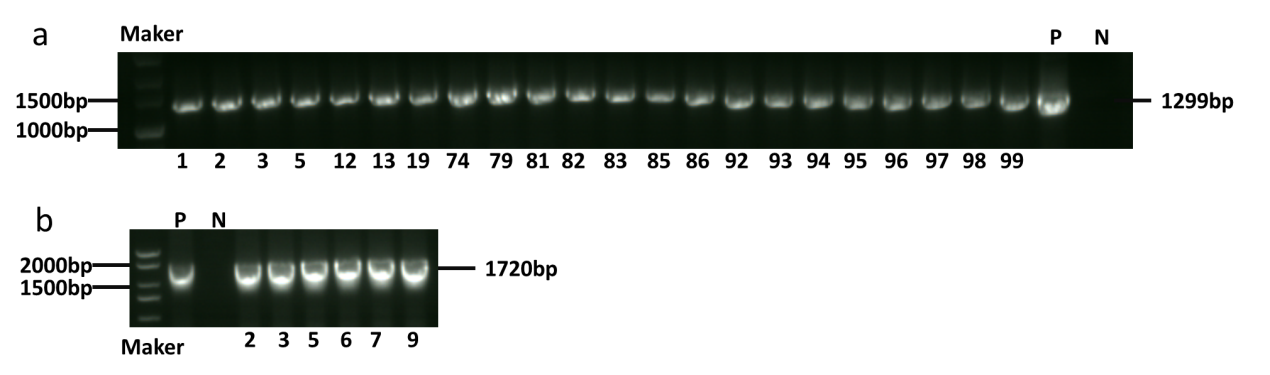


Fig. S8 PCR identification of transgene positive transformation events. (a) PCR detection of *GhABF3* transgenic Arabidopsis. (b) PCR detection of *GhABF3* transgenic cotton. P stands for positive control, the substrate is the 35S::GhABF3 plasmid, N stands for negative control, and the substrate is the DNA of the transformed recipient plant. In order to ensure the simplicity of the image, the gel image is appropriately cropped. The original image is attached to Figure S9.


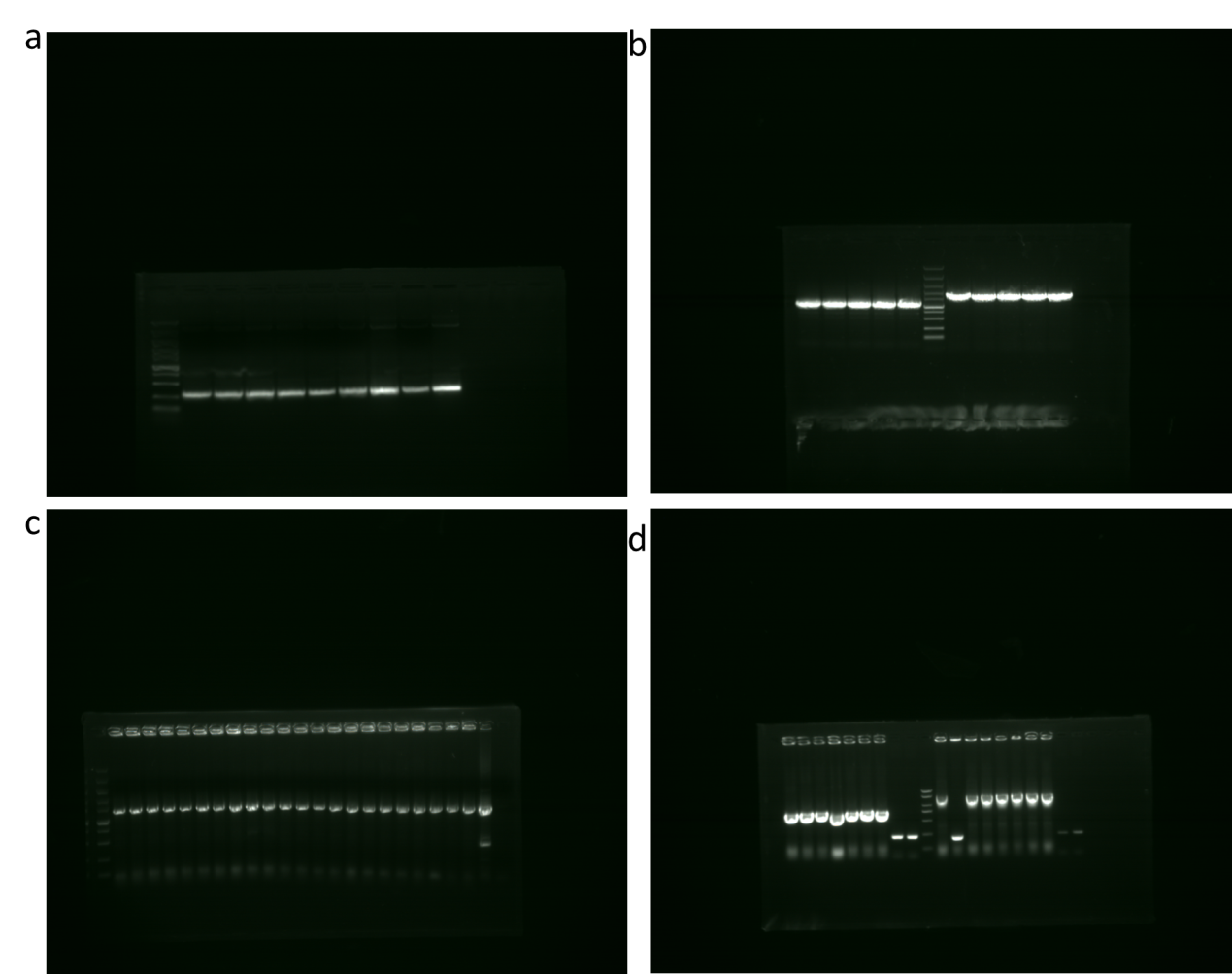


Fig. S9 Images of the original untreated gel used in Figures S5 and S8. (a) The original image used for Figure S5a. (b) The original image used in Figure S5b. On the left of marker is the used part in Figure S5b, and on the right is the band generated by other genes cloned at the same time. (c) The original image used in Figure S8a. (d) The original image used in Figure S8b. On the right of marker is the clipping part in Figure S8b, and on the left is the band generated by other genes simultaneously identified.


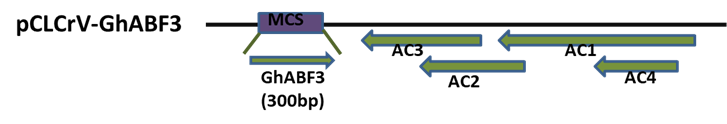


Fig. S10 The map of pCLCrV-GhABF3 vector. The arrow represents the direction of the insertion sequence. For more detailed carrier atlas information, please refer to [61] mentioned by predecessors.


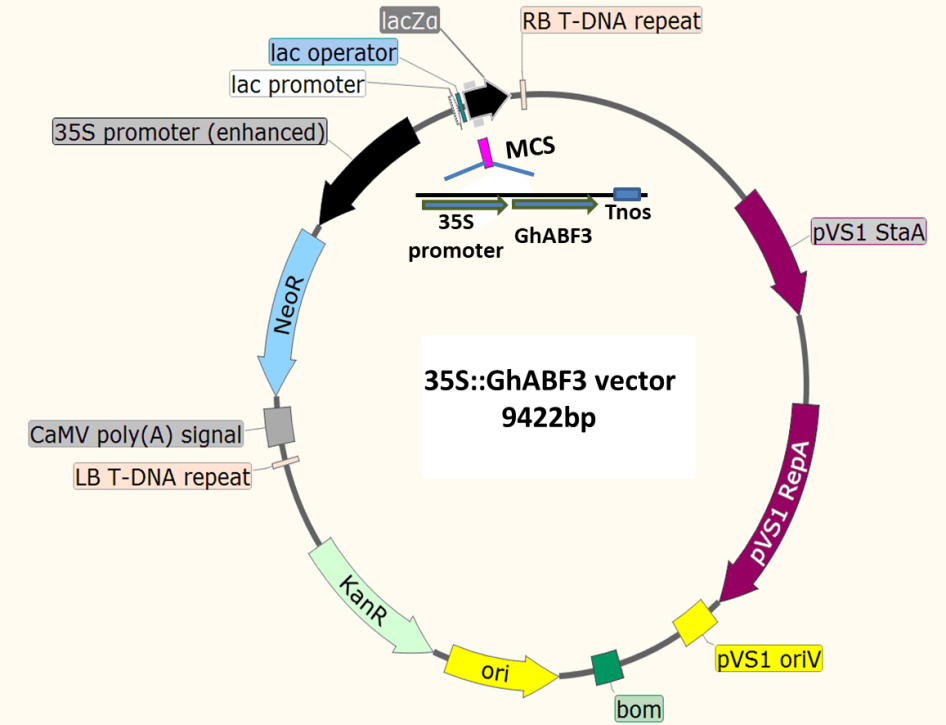


Fig. S11 The map of 35S::GhABF3 vector. The sequence of the green arrow inside the circle is the inserted sequence, and the direction of the arrow represents the direction of the inserted sequence.
